# 1888. The Impact of COVID-19 on Adherence to Directly Observed Therapy Among a Tuberculosis Treatment Cohort

**DOI:** 10.1093/ofid/ofad500.1716

**Published:** 2023-11-27

**Authors:** Victoria Overbeck, Samantha Malatesta, Tara Carney, Bronwyn Myers, Charles Parry, C Robert Horsburgh, Daniel Theron, Laura F White, Robin Warren, Karen R Jacobson, Tara C Bouton

**Affiliations:** Boston Medical Center, Brookline, Massachusetts; Boston University School of Public Health, Boston, Massachusetts; South African Medical Research Council, Cape Town, Western Cape, South Africa; Curtin University, Perth, Western Australia, Australia; South African Medical Research Council, Cape Town, Western Cape, South Africa; Boston University, Boston, Massachusetts; BREWELSKLOOF TB HOSPITAL, WORCESTER, Western Cape, South Africa; Boston University School of Public Health, Boston, Massachusetts; Stellenbosch University, Stellenbosch, Western Cape, South Africa; Boston University School of Medicine, Boston, Massachusetts; Boston Medical Center and Boston University School of Medicine, Boston, Massachusetts

## Abstract

**Background:**

The COVID-19 pandemic negatively impacted all levels of tuberculosis (TB) treatment services, including directly observed therapy (DOT) programs used to promote medication adherence. We compared the rate of adherence to DOT before and during the pandemic among observational research study participants on drug-susceptible (DS)-TB therapy in Worcester, South Africa.

**Methods:**

We analyzed adherence data from 263 study participants who participated in DOT for the duration of their DS-TB treatment. Participants enrolled between May 16, 2017, to September 27, 2019, and October 16, 2020, to March 4, 2022, were classified as pre- and post-COVID lockdown groups respectively. Adherence to DOT was used as a proxy for treatment adherence and defined as the number of successful DOT encounter days. Negative binomial regression models were used to compare the rate of non-adherence to DOT between the two groups. A sensitivity analysis was conducted that excluded participants in the post-COVID lockdown group enrolled after May 30, 2021, when South Africa increased their COVID-19 related restrictions in response to a third COVID wave (Figure 1).

**Results:**

The overall median rate of DOT adherence across both groups was 87.1% (IQR: 75.47%, 92.64%). The adjusted rate of non-adherence to DOT was higher in the post-COVID lockdown group (aIRR = 1.42, 95% CI = 1.04 – 1.96, p = 0.028) compared to the pre-COVID lockdown group, after adjusting for age, sex, employment status, household hunger, depression risk, and smoked illicit drug use. The rate of non-adherence to DOT was even greater in the post-COVID group (aIRR = 1.74, 95% CI = 1.17–2.67; p=0.006) when participants enrolled after May 30, 2021, were excluded from the analysis (Figure 2).

**Figure:**
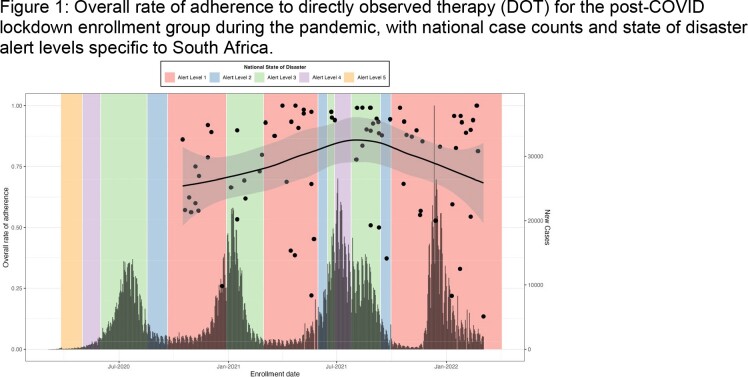

Solid lines are weighted least squares regression line with local fitting and 95% confidence intervals, implemented with R function geom_smooth.

**Figure:**
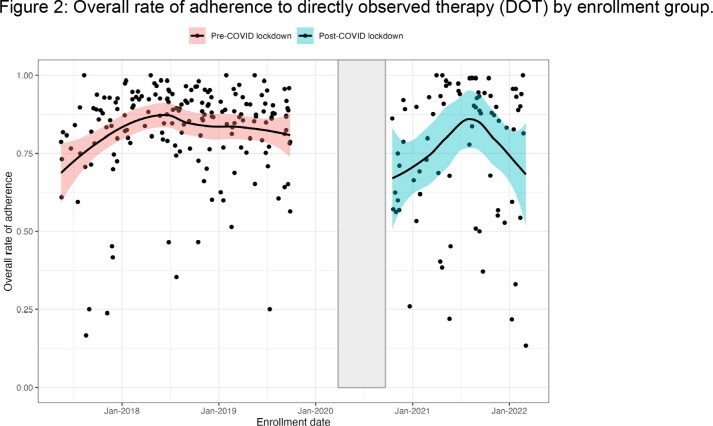

Solid lines are weighted least squares regression line with local fitting and 95% confidence intervals, implemented with R function geom_smooth. Rectangle represents period that study enrollment was paused due to COVID-19 restrictions (March 26, 2020 – September 20, 2020).

**Conclusion:**

The COVID-19 pandemic had significant adverse effects on participants’ adherence to a TB DOT programs in this setting. The change in DOT adherence persisted even after adjusting for measurable socioeconomic and behavioral variables and was most notable immediately following the first wave of COVID-19 infections. Further work is needed to understand barriers to treatment adherence that were exacerbated by the COVID-19 pandemic itself and lockdowns in order to identify individuals needing additional supports and ways to improve systems even during non-pandemic times.

**Disclosures:**

**All Authors**: No reported disclosures

